# Carotid artery dissection and brain herniation following traditional carotid massage: a case report

**DOI:** 10.1186/s12883-026-04820-w

**Published:** 2026-03-14

**Authors:** Abdullahi Ahmed Ahmed, Sahra Ali Yusuf

**Affiliations:** 1 Department of Emergency Medicine, Mogadishu Somali Turkey Training and Research Hospital, Mogadıshu, Somalia; 2 General Medicine and Surgery, Mogadishu Somali Turkey Training and Research Hospital, Mogadıshu, Somalia

**Keywords:** Carotid artery dissection, Neck manipulation, Ischemic stroke, Malignant middle cerebral artery infarction, Resource-limited settings, Case report

## Abstract

Carotid artery dissection is an important cause of ischemic stroke in young adults and has been associated with minor cervical trauma, including neck manipulation. Traditional carotid massage, commonly practiced in low-resource settings, is often perceived as harmless despite potential vascular risks. We report the case of an 18-year-old woman with no known comorbidities who developed acute neurological deterioration shortly after undergoing carotid massage by a traditional healer. Brain computed tomography revealed a large left hemispheric infarction involving the malignant middle cerebral artery territory, with associated cerebral edema, mass effect, and midline shift.

Due to rapid clinical deterioration culminating in cardiac arrest and death within hours of presentation, advanced vascular imaging was not performed. Therefore, a diagnosis of carotid artery dissection was considered presumptive, based on clinical presentation and temporal association. The clinical course was further complicated by respiratory compromise and suspected pneumonia in the context of preceding fever, as well as hemodynamic instability. Although decompressive hemicraniectomy was recommended, surgical intervention was declined due to contextual and resource-related factors.

This case highlights a severe ischemic stroke temporally associated with traditional carotid massage, while emphasizing the limitations in establishing causality in the absence of confirmatory vascular imaging. It underscores the need for cautious interpretation of such associations, increased public awareness regarding potentially harmful neck manipulation practices, and improved access to timely diagnostic and neurosurgical care in resource-limited settings.

## Introduction

Carotid artery dissection (CAD) is a recognized but often underdiagnosed cause of ischemic stroke in young adults, accounting for up to 20–25% of cases in patients under 45 years [1, 2]. It may occur spontaneously or after minor cervical trauma, including neck manipulation or massage [3]. The underlying mechanism involves intimal injury or intramural hematoma, leading to arterial stenosis, thromboembolism, or occlusion [4].

Traditional healing practices are widely used in low-resource settings, including East Africa, where access to formal healthcare may be limited [5]. Neck massage by traditional healers is commonly performed for nonspecific symptoms but may expose patients to vascular injury, particularly involving the carotid arteries [6]. The carotid bulb is especially vulnerable to external mechanical stress [7]. Although prior studies have reported an association between cervical manipulation and CAD, data from African settings remain limited [8, 9].

Malignant middle cerebral artery (MCA) infarction is a severe complication of large-vessel stroke, characterized by extensive edema, mass effect, and high mortality [10, 11]. Early recognition and management, including antithrombotic therapy and decompressive hemicraniectomy, are critical but may be limited in resource-constrained settings [1, 12].

We report a young woman with a large hemispheric infarction following traditional neck massage. In the absence of vascular imaging, CAD is considered a presumed diagnosis based on clinical and temporal association. This case highlights a possible link between traditional neck manipulation and severe stroke, while emphasizing diagnostic uncertainty and the need for increased awareness in similar settings.

## Case report

An 18-year-old woman with no known chronic medical illnesses presented to the emergency department with a five-day history of fever, one day of altered consciousness, and a two-day history of right-sided hemiplegia. According to her family, she had undergone carotid massage by a traditional healer shortly before the onset of confusion and neurological deterioration. It is unclear whether the massage was sought in response to the fever or other nonspecific symptoms.

On arrival, she was unconscious with a Glasgow Coma Scale (GCS) score of 7. Her pupils were mid-dilated and sluggishly reactive to light. Respiratory examination revealed decreased air entry over the right lung field, with oxygen saturation of 85% on room air. Vital signs showed blood pressure of 117/69 mmHg, pulse rate of 81 beats per minute, and random blood glucose of 83 mg/dL. Neurological examination confirmed right-sided hemiplegia.

Laboratory investigations demonstrated leukopenia, lymphopenia, and anemia, with elevated C-reactive protein levels and deranged liver and renal function tests. Arterial blood gas analysis revealed metabolic acidosis as the primary acid–base disorder.

**Fig. 1:**
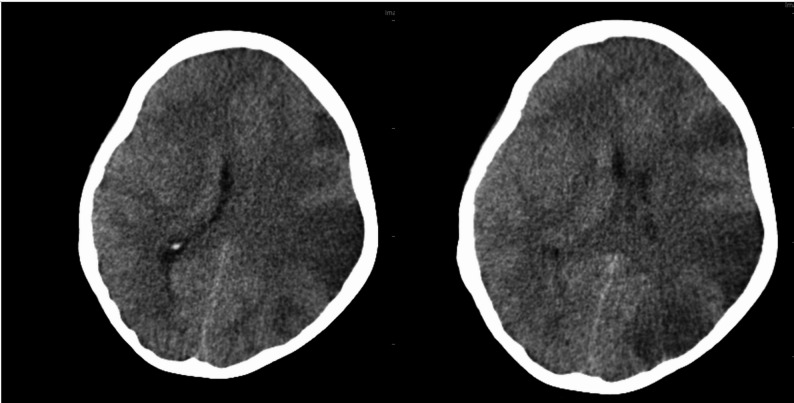
A large, ill-defined hypoattenuation within the left cerebral hemisphere demonstrates mass effect, manifesting as mild ipsilateral lateral ventricular effacement and left mild uncal herniation, highly suggestive of extensive ischemic insult

**Fig. 2:**
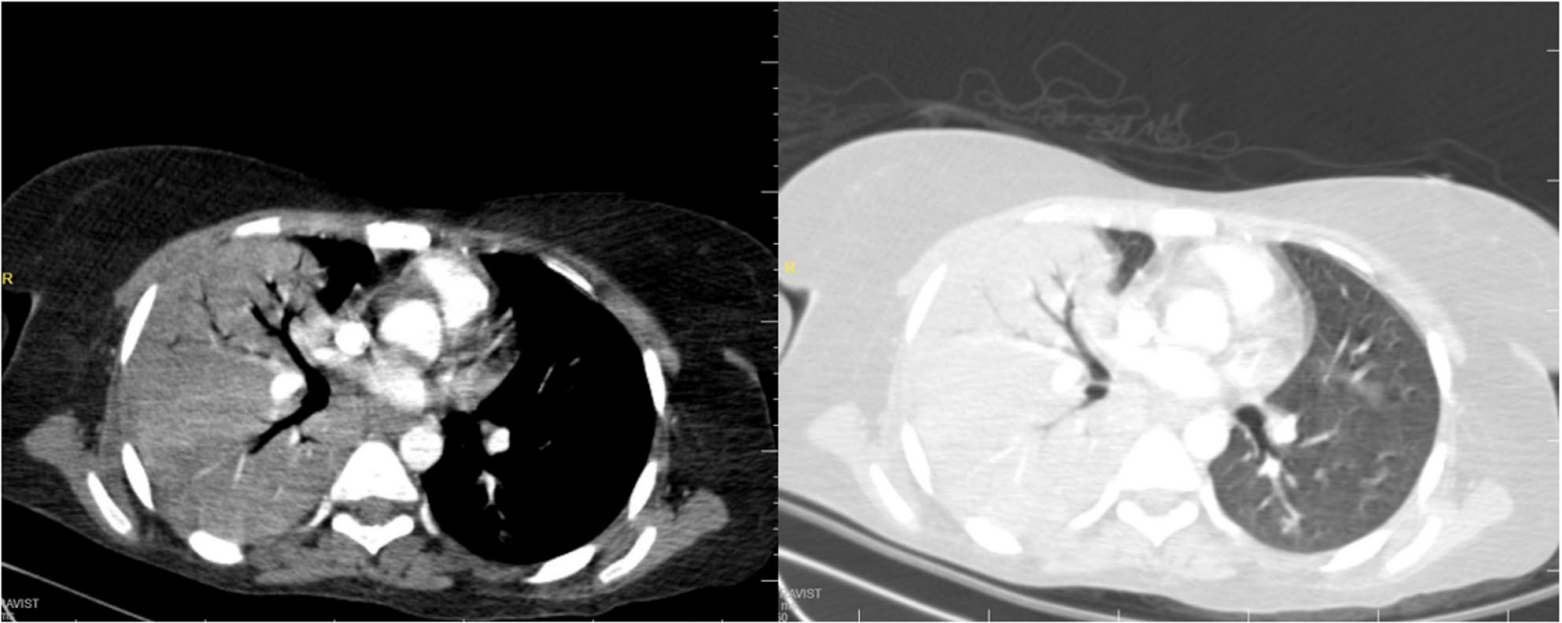
Chest computed tomography demonstrating extensive aspiration pneumonia, characterized by confluent consolidation with prominent air bronchograms involving the majority of the right lung, associated with a small right-sided pleural effusion. Additional discrete areas of consolidation are noted within the left upper and lower lobes

Non-contrast computed tomography (CT) of the brain showed diffuse cerebral edema with features of a large left middle cerebral artery (MCA) infarction, associated with a 3 mm midline shift and compression of the frontal and occipital horns of the left lateral ventricle (Fig. 1). Chest CT revealed a homogeneous opacity in the right lung, consistent with pneumonia, which may reflect aspiration or a community-acquired process in the context of preceding fever (Fig. 2).

Due to rapid clinical deterioration culminating in cardiac arrest and death within hours of presentation, advanced vascular imaging, such as CT angiography or Doppler ultrasound, could not be performed. The absence of imaging was related to the patient’s unstable condition, limited time for intervention, and constraints of the resource-limited setting. Therefore, carotid artery dissection was considered a presumed diagnosis based on clinical presentation and temporal association. The clinical course was further complicated by respiratory compromise and suspected pneumonia in the context of preceding fever, as well as hemodynamic instability.

During observation, the patient acutely deteriorated and suffered a cardiac arrest, from which she was successfully resuscitated. Given evidence of significant mass effect and impending herniation, urgent decompressive hemicraniectomy was recommended; however, the family declined surgical intervention due to contextual and resource-related factors, including logistical constraints and cultural considerations. Medical management with intravenous mannitol was initiated, and the patient was admitted to the intensive care unit (ICU) for close monitoring.

While in the ICU, she developed hemodynamic instability and experienced a second cardiac arrest. Resuscitation efforts were ongoing at the time of reassessment. Empirical broad-spectrum antibiotic therapy was started for presumed pneumonia and sepsis, including aztreonam 2.5 g intravenously three times daily and metronidazole 500 mg intravenously three times daily. Blood cultures were obtained, and supportive care was continued. Antiplatelet or anticoagulation therapy was not initiated due to the severity of infarction, cerebral edema, and hemodynamic instability.

This case illustrates a severe ischemic stroke temporally associated with traditional carotid massage. It underscores the limitations in establishing causality without confirmatory vascular imaging, highlights the challenges of managing large-vessel stroke in resource-limited settings, and emphasizes the need for increased awareness of potential risks related to unregulated neck manipulation. The report also highlights challenges in timely access to neurosurgical intervention and the importance of careful clinical interpretation in similar contexts.

## Discussion

Carotid artery dissection (CAD) is an important cause of ischemic stroke in young adults and may be precipitated by minor cervical trauma, including neck manipulation or massage [1, 2]. In this case, the temporal association between traditional carotid massage and abrupt neurological deterioration raises the possibility of a contributing role; however, causality cannot be established, as vascular imaging was not performed. The patient’s rapid clinical decline and the constraints of the resource-limited setting precluded CT angiography or Doppler ultrasound, and therefore CAD remains a presumed diagnosis based on clinical presentation and timing. Biomechanical stress applied to the cervical vasculature may theoretically predispose to intimal injury or intramural hematoma formation, which can lead to arterial stenosis, thromboembolism, or occlusion, potentially resulting in large territorial cerebral infarction [4, 13].

Traditional healing practices remain widely utilized in East Africa and other low-resource settings, often outside formal regulatory frameworks and without standardized training or risk disclosure [5]. While vertebral artery dissection following cervical manipulation has been more commonly reported, carotid artery dissection after massage or manual neck therapy is less frequently documented in African literature [6, 8]. The apparent scarcity of reported cases likely reflects underrecognition, delayed presentation, and limited access to advanced neuroimaging, rather than true rarity. This case therefore provides regional evidence highlighting a potential, preventable contributor to severe neurological injury.

The evolution to malignant middle cerebral artery (MCA) infarction in this patient represents a severe complication of large-vessel stroke. Malignant MCA infarction is characterized by rapid cytotoxic edema, mass effect, midline shift, and risk of transtentorial herniation, with high mortality if timely surgical intervention is not feasible [10, 14]. Early decompressive hemicraniectomy can significantly reduce mortality and improve functional outcomes, particularly in younger patients [15]. In this case, refusal of surgical decompression by the family reflects the influence of cultural beliefs, health literacy, and resource constraints on critical care decisions in low-resource settings.

The patient’s clinical course was further complicated by fever preceding neurological deficits, respiratory compromise, suspected pneumonia, metabolic derangements, and multiorgan dysfunction. These factors are well-established contributors to poor outcomes in severe ischemic stroke [16]. Given the preceding fever, the possibility of community-acquired pneumonia should be considered, alongside aspiration as a consequence of depressed consciousness. These intercurrent complications likely contributed to recurrent cardiac arrests and clinical deterioration.

Laboratory abnormalities—including leukopenia, anemia, and derangements in liver and renal function—reflect systemic illness and may have influenced the patient’s vulnerability to secondary complications. Antiplatelet or anticoagulation therapy was not administered due to the presence of large infarction, cerebral edema, and hemodynamic instability.

This case underscores several important clinical and public health considerations. First, CAD should be considered in young patients presenting with acute ischemic stroke, particularly in the context of recent minor cervical trauma or manipulation, while recognizing diagnostic limitations in resource-constrained settings [17]. Second, public health education is needed to raise awareness about the potential risks of unregulated neck manipulation. Finally, improving access to early neuroimaging, stroke expertise, and timely neurosurgical intervention is essential to mitigate stroke-related morbidity and mortality in East Africa and similar low-resource environments [18].

### Limitations

This report is limited by the absence of confirmatory vascular imaging, the inability to definitively establish causality between carotid massage and stroke, and the single observational nature of the case. These limitations should be considered when interpreting the findings.

## Conclusion

This case illustrates a severe ischemic stroke temporally associated with traditional carotid massage in a young adult, with presumed carotid artery dissection based on clinical presentation and timing. While causality cannot be definitively established in the absence of vascular imaging, the case highlights the potential risks of unregulated neck manipulation. It underscores the importance of early recognition of cervical arterial injury in young patients presenting with acute stroke, heightened clinical vigilance, and timely access to neuroimaging and neurosurgical intervention. Public education and improved healthcare resources are essential to reduce preventable morbidity and mortality in similar resource-limited settings.

## Data Availability

We confirm that we have complete access to all data in this study and accept full responsibility for its integrity. Data supporting the findings of this study are available upon reasonable request from the corresponding author.
